# A multilevel model of life satisfaction among old people: individual characteristics and neighborhood physical disorder

**DOI:** 10.1186/s12889-019-7228-9

**Published:** 2019-07-03

**Authors:** Camila Teixeira Vaz, Amanda Cristina de Souza Andrade, Fernando Augusto Proietti, César Coelho Xavier, Amélia Augusta de Lima Friche, Waleska Teixeira Caiaffa

**Affiliations:** 1 Department of Physical Therapy, Federal University of Juiz de Fora - Campus Governador Valadares, Rua São Paulo 745, Governador Valadares, 35010-180 Brazil; 2 Faculty of Medicine, Federal University of Minas Gerais, Avenida Alfredo Balena 190, Belo Horizonte, 30130-100 Brazil; 3 Belo Horizonte Observatory for Urban Health, Avenida Alfredo Balena 190, Belo Horizonte, 30130-100 Brazil; 4 Institute of Public Health, Federal University of Mato Grosso, Avenida Fernando Corrêa 2367, Cuiabá, 78060-900 Brazil; 5 Faculty of Health and Human Ecology, Rua São Paulo 958, Vespasiano, 33200-000 Brazil

**Keywords:** Aged, Built environment, Urban health, Multilevel analysis

## Abstract

**Background:**

Considering the lack of studies that examine built environmental factors associated with life satisfaction among old people in developing countries, particularly those focused on Brazil, the aim of this study was to estimate the prevalence of life satisfaction among old adults residents in a Brazilian urban center and to investigate its association with individual characteristics and objective measures of the built environment.

**Methods:**

A household survey (*N* = 832) in Belo Horizonte, Minas Gerais, Brazil (2008–2009) and a Systematic Social Observation (SSO) was used in this study. Life satisfaction was assessed through Self-Anchoring Ladder Scale, developed by Cantril, in 1965. Participants’ answers were categorized as satisfied (rungs 6–10) and dissatisfied (rungs 0–5). A Multilevel Poisson regression analysis with robust variance was performed.

**Results:**

The prevalence of satisfaction with life was approximately 82%. Higher prevalence of life satisfaction was significantly associated with old people who reported higher incomes, higher religious participation, who practice physical activity and who perceive their health as good and very good. In contextual level, results showed that when the contextual features were adjusted separately by the individual characteristics they were no longer significant. The results also showed a lower prevalence of life satisfaction among those living in neighborhoods with higher physical disorder, even after adjusting for individual and other contextual characteristics.

**Conclusions:**

The present findings suggest that life satisfaction should be assessed whenever evaluating urban redevelopment programs designed to improve neighborhood characteristics, reducing physical disorder, especially among old adults.

## Background

Life satisfaction is a global assessment of one’s life [1, 2]. According to the Organization for Economic Cooperation and Development [3] life satisfaction “measures how people evaluate their lives as a whole rather than their current feelings”. This term is often used interchangeably with happiness and subjective wellbeing in many studies around the world [4].

Life satisfaction closely relates to health [5], as an important predictor of mortality, morbidity, depression, and health status over the life course [6–9]. This link is especially pronounced in older people because the aging period is frequently accompanied by illnesses, disabilities, and dependency for care and support [5, 10]. In addition, life satisfaction is considered an essential component of successful aging. There are ongoing research efforts to identify its associated factors that may help to reach the ideal aging model [11].

In old population, studies carried out in developed countries have identified some individual-level factors related to life satisfaction. They are demographic and socio-economic factors, lifestyle behavior, health status, social activities and connections. Regarding contextual-level factors, studies tend to be exclusively focused on the role of the social environment, such as social support and social interaction [5, 12–16].

Recently, besides social environment, ecological theories of aging suggest that as people age and their functional capacity declines, the built (or physical) environment in which they live holds an emerging role in their life satisfaction [17, 18]. Urban built environment for senior residents may be a relevant promising strategy for improving old people’s life satisfaction, with subsequent successful aging in this setting [19]. However, neither built environment nor life satisfaction in this population has been largely examined [20], especially in developing countries.

Considering the lack of studies that examine built environmental factors associated with life satisfaction among old populations in urban Latin America, particularly those focused on Brazil, the aim of this study was to estimate the prevalence of life satisfaction among old adults residents in a Brazilian urban center and to investigate its association with individual characteristics and objective measures of the built environment.

## Methods

### Study design

Data were obtained from a multi-method epidemiological study that included a household survey, called BH Health Study (Saúde em Beagá, in Portuguese language) and an objective environmental characterization using Systematic Social Observation (SSO), both carried out by the Observatory for Urban Health in Belo Horizonte (OSUBH) at the Federal University of Minas Gerais. Two of the nine health districts of Belo Horizonte (Oeste and Barreiro) were included in this study, which together account for 24% of the total of residents of the municipality [21]. Health districts comprise a geographical area that includes a population with epidemiological and social characteristics, their needs, and the health resources to attend it. These health districts were selected due to field research logistics and their internal heterogeneity in terms of select demographic, socioeconomic, and health indicators [22].

The BH Health Study (2008–9), included adults residing in households, through a probabilistic sampling design, stratified and clustered, in three stages: census tract (*n* = 149); household (*n* = 4048); and a randomly selected adult resident (18 years or older) within eligible households, with the total of 4048 participants. Individuals aged 60 years or older (*n* = 834) were included in the present study analysis. Details on the survey have been published previously [22].

SSO was used to assess and quantify the built environmental characteristics associated with health-related events of the studied neighborhood. Observations were conducted between April and June of 2011 in the same geographical area of the BH Health Study by independent observers. The units of analysis were street segments within a 100- m range, in any direction, from the households of the survey’s participants. Segments in the same census sector were considered the neighborhood corresponding to that census tract. The final sample included 1295 segments. Instrument reliability was evaluated in a previous study and was adequate for the observation of characteristics with greater temporal stability, mainly regarding services, pedestrian environment, and safety [23]. Details on the method have been published previously [23, 24].

All subjects gave their informed consent for inclusion before they participated in the study. The study was conducted in accordance with the Declaration of Helsinki, and the protocol was approved by the Ethics Committee of Federal University of Minas Gerais (protocol n. ETIC 253/06) and the Ethics Research Committee of the Department of Municipal Health (073.2008).

### Outcome

The response variable of life satisfaction was measured using the Self-Anchoring Ladder Scale (SALS), developed by Cantril [25]. This instrument consists of a scale from 0 to 10 points, represented schematically by a ladder, in which the lowest rung indicates the lowest satisfaction with life and the highest rung the highest. Participants were asked to answer the following question: “In relation to satisfaction with your current life, in which rung are you TODAY?” In order to carry out the analysis, participants’ answers were categorized as satisfied (rungs 6–10) and dissatisfied (rungs 0–5), as in a previous study [26].

### Explanatory variables

Individual and contextual level explanatory variables were used according to the source of information (survey or SSO). Individual variables were selected considering the literature review [5, 14, 15, 27–29] and they included demographic and socio-economic variables, participation in religious activities, lifestyle and health status.

Demographic and socio-economic variables were: age (years), sex (female and male), marital status (with partner; widower; and without partner), schooling (0 to 4; 5 to 8; 9 to 11; and 12 or more years of formal education), employment situation (never worked; currently working; and has worked before), and family income as a multiple of the minimum monthly wage (< 2 minimum monthly wages; ≥ 2 minimum monthly wages). Religion participation was assessed by the frequency of participation in religious services (none; ≤ twice a month; > twice a month). Lifestyle variables were: physical activity (yes or no), assessed by the question: “Do you practice (or have you practiced) any physical activity in the past 3 months?” [30]; smoking habits (non-smoker; former smoker; and current smoker), constructed from the following questions: “In your life, have you ever smoked cigarettes?” and “Do you currently smoke cigarettes?” [30]; and alcohol consumption (yes or no), assessed by the question: “Do you drink alcoholic beverages?” [30].

Finally, health status variables were: health services use (yes or no), assessed by the question: “Did you seek services or any professional appointment for your health care in the past 30 days?”; morbidity (none; one; and two or more illness), assessed by the question: “At any time, has a doctor or other health care professional ever said that you have any of the illness listed below: hypertension, high cholesterol, diabetes, asthma, arthritis, arthrosis, rheumatism, osteoporosis, chronic kidney disease, depression, migraines, epilepsy, tuberculosis, cancer, heart disease, lung disease, chronic digestive disease (ulcer/gastritis), or mental illness?”; and self-rated health (very good/good; fair; and poor/very poor), assessed by the question: “In general, would you say that your health is: very good, good, fair, poor, or very poor?”

Contextual level variables were obtained from composite indicators, constructed using a principal component analysis via the covariance matrix, which was based on the information collected through SSO and aggregated by census tract. First, simple indicators were structured and then these indicators were grouped in domains from which composite indicators were respectively developed. The internal consistency of the proposed domains was considered acceptable, with Cronbach’s alpha ranging from 0.591 to 0.820. Details on the indicator development process have been published recently [24].

This present study included the following SSO domains: *Street conditions and traffic infrastructure items*; *Walking environment*; *Accessibility*; *Spaces for physical activity and leisure*; *Physical disorder*; *Safety*; as well as the presence of Services, subdivided in *Food, health and recreational services*; *Garbage collection and school services* and *Automotive mechanics and repair services*. The *Street conditions and traffic infrastructure items* scale was developed based on the evaluation of the following items: public transport signage; prohibited parking signs; and presence of flowerbeds, speed bumps, radars, and traffic lights. The *Walking environment* scale included evaluations of items such as: sidewalk paving; trees for shading; sidewalk width at the smaller end; and favorable perception for walking. The *Accessibility* domain was developed based on the evaluation of the items: access ramps or tactile floors; and pedestrian traffic items (grids, crosswalks, walkways). The *Spaces for physical activity and leisure* scale included items regarding the evaluation and presence of spaces for physical activity; of parks and plazas; and favorable perceptions of the environment for physical activity. The *Physical disorder* domain was composed by the following items: presence of trash (needles, cigarettes, cans and condoms) and graffiti in public urban equipments/facilities. The *Safety* scale was developed from the variables: public lighting; policing; and safety items in buildings/properties (either observed by the presence of the item or by a safety note informing the presence of dogs, alarms, wires, gate/pointed walls, windows with grids, electric fences, doormen, glass shards, and camera surveilance). The items of the *Food, health and recreational services* scale referred to the presence of: vendors of fresh and locally-grown food, convenience stores, vegetable vendors, private health care services, and public or private recreation facilities. The items of the *Garbage collection and school services* scale referred to the presence of: public or private garbage collectors; and elementary and high schools. Last, the items of the *Automotive mechanics and repair services* scale referred to the presence of: mechanical workshops and automotive repair centers. The scales range from 0 to 5, and a higher domain score denotes a greater presence of the attribute in the neighborhood.

### Statistical analysis

Descriptive analysis was performed through frequency distributions, averages and standard deviation (SD). The prevalence of life satisfaction at a 95% confidence interval (95%CI) was estimated for the population sample and for each of the individual variables. The means and SD of the contextual variables (*Street conditions and traffic infrastructure items*; *Walking environment*; *Accessibility*; *Spaces for physical activity and leisure*; *Physical disorder*; *Safety*; *Food, health and recreational services*; *Garbage collection services and school*; and *Automotive mechanics and repair services*) were calculated and stratified by the satisfied and dissatisfied condition. All the contextual variables were considered protective factors for satisfaction with life, with the exception of the Physical Disorder considered risk factor.

To verify the association between life satisfaction and explanatory variables in the bivariate and multivariate analysis, the multilevel Poisson regression with robust variance was used. Robust Poisson regression was used because the outcome (Satisfied) had a prevalence of greater than 10%, causing the odds ratio to deviate from the true risk ratio [31]. Initially, a null model was adjusted to evaluate the contextual effects. Then, bivariate models were adjusted for individual and contextual variables. Finally, multivariate models were adjusted. The first one included individual variables whose *p*-value was equal to or smaller than 0.20 in the bivariate analysis. For this model, the stepwise-backward procedure was used to select the variable to retain (*p* ≤ 0.05), except for age and sex, used as adjustment variables. Then, by adding the individual variables that remained in the multivariate model, one model was adjusted for each contextual variable with a *p*-value ≤0.20 in the bivariate analysis. The last model was adjusted with all individual and contextual variables selected in the previous steps. In this step, multiplicative interactions between the contextual variables included in the multivariate model were tested. All multilevel analyses were performed using a fixed effects model with a random intercept and log function to obtain the prevalence ratio (PR) and 95%CI measures. The Akaike information criterion (AIC) was used to assess the appropriateness of model. Finally, the Spearman coefficient was used to estimate the correlations between contextual variables.

The software Stata, version 12.0 (StataCorp LP, College Station, USA) was used. All analyses were carried out taking the complex sample into account. Significance was set at 5%, with a 95%CI.

## Results

Of the total 834 study participants, two were excluded due to lack of information on the outcome variable. The total number of census tracts analyzed in this study was 146, since one census tract lacked old adults residents from which to select participants. The mean number of old participants per census tract was 6, ranging from 1 to 15.

The prevalence of satisfied individuals was 81.95% (95% CI: 78.66–85.24); 56.41% were women and the mean age was 69.29 (SD = 7.68). About 61% of old people reported to live with a partner, 51% had from 0 to 4 years of formal education; 77% reported having a family income of ≥2 minimum monthly wages. Bivariate analysis showed a positive dose-response between schooling and life satisfaction; that is, as the number of years of school education increases, there is an increase in the prevalence of old people who are satisfied with life. The prevalence of life satisfaction was higher among old people with a family income of ≥2 minimum monthly wages, those who practiced physical activity, those who never smoked and, those who perceived their health as good/very good or fair. The variables age, sex, marital status, employment situation, frequency of participation in religious services, alcohol consumption, health service use, and morbidity were not associated with life satisfaction in old people (Table 1).

**Table 1:** Univariate analysis of life satisfaction and individual characteristics among old people. The BH Health Study, Belo Horizonte, Minas Gerais State, Brazil, 2008–2011

Variables	TotaL	life satisfaction		PR (95% CI)	*p*-value
	N = 832	Satisfied	Dissatisfied		
Age m(SD)	69.29 (7.68)	69.54 (7.74)	68.18 (7.29)	1.02 (0.99–1.05)^1^	0.138
Sex (%)					
Male	43.59	80.52	19.48	0.97 (0.89–1.05)	0.446
Female	56.41	83.05	16.95	1.00	
Marital status (%)					
With partner	61.11	81.81	18.19	1.01 (0.90–1.14)	0.814
Widower	23.19	83.19	16.81	1.03 (0.90–1.18)	0.653
Without partner	15.7	80.66	19.34	1.00	
Schooling (%)^a^					
12 or more years	16.2	89.19	10.81	1.15 (1.04–1.27)	0.006
9 to 11 years	19.58	87.14	12.86	1.13 (1.02–1.25)	0.025
5 to 8 years	13.27	82.79	17.21	1.07 (0.93–1.27)	0.339
0 to 4 years	50.95	77.43	22.57	1.00	
Employment situation (%)					
Currently working	22.84	77.76	22.24	0.90 (0.80–1.02)	0.296
Has worked before	62.26	82.46	17.54	0.96 (0.86–1.06)	0.404
Never worked	14.91	86.20	13.80	1.00	
Family income (%)^b^					
≥ 2 mw	77.21	84.50	15.50	1.15 (1.04–1.28)	0.007
< 2 mw	22.79	73.21	26.79	1.00	
Religion participation (%)^**a**^					
≤ 2 x/month	29.83	80.17	19.83	1.17 (0.99–1.38)	0.067
> 2 x/month	58.54	84.74	15.26	1.11 (0.93–1.32)	0.258
None	11.63	72.41	27.59	1.00	
Physical activity (%)					
Yes	57.86	87.84	12.16	1.13 (1.03–1.24)	0.010
No	42.14	77.66	22.34	1.00	
Smoking habits (%)					
Non-smoker	51.72	84.30	15.7	1.20 (1.01–1.44)	0.041
Former smoker	37.96	82.00	18.00	1.17 (0.98–1.40)	0.080
Current smoker	10.32	69.97	30.03	1.00	
Alcohol consumption (%)					
Yes	29.51	82.22	17.78	1.00 (0.92–1.10)	0.921
No	70.49	81.83	18.17	1.00	
Health service use (%)					
Yes	37.31	79.93	20.07	0.96 (0.88–1.05)	0.395
No	62.69	83.15	16.85	1.00	
Morbidity (%)					
None	9.7	89.28	10.72	1.11 (0.99–1.24)	0.085
One	18.38	83.29	16.71	1.03 (0.91–1.17)	0.606
Two or more	71.92	80.62	19.38	1.00	
Self-rated health (%)					
Good/Very good	50.86	85.59	14.41	1.29 (1.07–1.55)	0.007
Fair	36.6	82.20	17.80	1.24 (1.01–1.51)	0.033
Poor/Very poor	12.46	66.31	33.69	1.00	

*m* Mean, *SD* standard deviation

^a^1 missing; ^b^30 missing; ^1^For an increase of 5 years in age

Regarding contextual characteristics (Table 2), the *Walking environment* scale was the most positively evaluated, with a mean of 2.95 (SD = 0.92). Contrarily, the *Spaces for physical activity and leisure* (mean = 0.65; SD = 0.94) and *Food, health and recreational services* (mean = 0.65; SD = 0.43) were the scales with the lowest evaluation, indicating the low frequency of these attributes in the neighborhood. Bivariate analysis showed higher prevalence of life satisfaction reported among older adults living in places with greater presence of items of *Walking environment*; *Safety*; and *Garbage collectors and school services*. *Street conditions and traffic infrastructure items*; *Accessibility*; *Space for physical activity and leisure*; *Physical disorder*; *Food, health and recreational services*; and *Automotive mechanics and repair services* scales were not associated with life satisfaction in the univariate analysis in this population.

**Table 2:** Univariate analysis of life satisfaction and contextual characteristics among old people. The BH Health Study, Belo Horizonte, Minas Gerais State, Brazil, 2008–2011

Variables	TotaL	Life Satisfaction		PR (95% CI)	*p*-value
	*N* = 146	Satisfied	Dissatisfied		
Street conditions and traffic infrastructure items m(SD)	0.81 (0.89)	0.93 (0.89)	0.90 (0.87)	1.01 (0.97–1.05)	0.762
Walking environment m(SD)	2.76 (1.04)	2.98 (0.93)	2.78 (0.82)	1.05 (1.01–1.08)	0.008
Accessibility m(SD)	1.13 (0.74)	1.26 (0.90)	1.26 (0.92)	1.00 (0.96–1.04)	0.980
Spaces for physical activity and leisure m(SD)	0.65 (0.99)	0.65 (0.96)	0.58 (0.88)	1.01 (0.98–1.05)	0.423
Physical disorder m(SD)	1.86 (0.77)	1.72 (0.72)	1.86 (0.79)	0.95 (0.90–1.00)	0.082
Safety m(SD)	1.46 (1.06)	1.75 (1.12)	1.50 (1.07)	1.04 (1.00–1.07)	0.026
Food, health and recreational services m(SD)	0.63 (0.47)	0.65 (0.41)	0.66 (0.49)	0.99 (0.92–1.07)	0.775
Garbage collection and school services m(SD)	1.26 (0.81)	1.30 (0.77)	1.16 (0.70)	1.04 (1.01–1.08)	0.020
Automotive mechanics and repair services m(SD)	2.26 (0.58)	2.28 (0.64)	2.26 (0.55)	1.01 (0.96–1.06)	0.680

*m* Mean, *SD* standard deviation

For the multivariate analysis six models were estimated as shown in Table 3. Model 1, consisting of only individual-level variables, suggested a higher prevalence of life satisfaction among old people with a family income of at least twice the minimum monthly wage (PR: 1.13; 95% CI: 1.02–1.26), among those who participated in religious services more than twice a month (PR: 1.20, 95% CI: 1.02–1.42), among those who practiced physical activity (PR: 1.12, 95% CI: 1.02–1.22), and among those who perceived their health as good/very good (RP: 1.23, 95% CI: 1.03–1.46). After adjustments for individual variables in Model 1, none of the contextual scales for *Walking environment*; *Physical disorder*; Safety; and *Garbage collection and school services* scales (models 2 to 5) were significantly associated with life satisfaction. However, significant associations were noted for the *Physical disorder* scale, in model 6 (PR: 0.94, 95% CI: 0.88–0.99). This model shows that old people living in census tracts with greater physical disorder reported less satisfaction with life. Finally, none of the interactions terms between the contextual variables tested were statistically significant.

**Table 3:** Multilevel models of life satisfaction, individual and contextual characteristics among old people. The BH Health Study, Belo Horizonte, Minas Gerais State, Brazil, 2008-2011

Variables	Model 1		Model 2		Model 3		Model 4		Model 5		Model 6	
	PR (95% CI)	*p*-value	PR (95% CI)	*p*-value	PR (95% CI)	*p*-value	PR (95% CI)	*p*-value	PR (95% CI)	*p*-value	PR (95% CI)	*p*-value
Individual characteristics												
Age	1.03 (1.01–1.06)^1^	0.013	1.03 (1.01–1.06)^1^	0.015	1.03 (1.01–1.06)^1^	0.010	1.03 (1.01–1.06)^1^	0.019	1.03 (1.01–1.06)^1^	0.014	1.03 (1.01–1.06)^1^	0.015
Sex												
Male	0.94 (0.87–1.02)	0.170	0.94 (0.87–1.02)	0.165	0.94 (0.87–1.02)	0.152	0.95 (0.87–1.03)	0.193	0.95 (0.87–1.03)	0.176	0.95 (0.87–1.03)	0.202
Female	1.00		1.00		1.00		1.00		1.00		1.00	
Family income												
≥ 2 mw	1.13 (1.02–1.26)	0.024	1.12 (1.00–1-26)	0.051	1.13 (1.01–1.26)	0.028	1.12 (1.01–1.26)	0.048	1.13 (1.01–1.26)	0.034	1.11 (0.98–1.25)	0.088
< 2 mw	1.00		1.00		1.00		1.00		1.00		1.00	
Religion participation												
> 2 x/month	1.20 (1.02–1.42)	0.027	1.20 (1.02–1.42)	0.027	1.20 (1.02–1.43)	0.025	1.21 (1.02–1.42)	0.026	1.20 (1.02–1.42)	0.019	1.21 (1.03–1.44)	0.022
≤ 2 x/month	1.14 (0.95–1.37)	0.147	1.14 (0.95–1.37)	0.153	1.14 (0.95–1.37)	0.152	1.15 (0.96–1.37)	0.141	1.14 (0.95–1.37)	0.149	1.15 (0.95–1.38)	0.143
None	1.00		1.00		1.00		1.00		1.00		1.00	
Physical activity												
Yes	1.12 (1.02–1.22)	0.019	1.11 (1.02–1.22)	0.022	1.11 (1.02–1.22)	0.018	1.11 (1.02–1.22)	0.020	1.11 (1.02–1.22)	0.021	1.11 (1.01–1.21)	0.023
No	1.00		1.00		1.00		1.00		1.00		1.00	
Self-rated health												
Good/Very good	1.23 (1.03–1.46)	0.006	1.22 (1.03–1.45)	0.021	1.23 (1.04–1.46)	0.018	1.22 (1.03–1.46)	0.025	1.23 (1.03–1.46)	0.020	1.21 (1.02–1.44)	0.026
Fair	1.20 (1.00–1.45)	0.051	1.20 (1.00–1.45)	0.055	1.20 (1.00–1.45)	0.047	1.20 (0.99–1.45)	0.056	1.20 (1.00–1.45)	0.052*	1.20 (1.00–1.44)	0.052
Poor/Very poor	1.00		1.00		1.00		1.00		1.00		1.00	
Contextual characteristics												
Walking environment			1.02 (0.98–1.06)	0.444							0.99 (0.95–1.04)	0.741
Physical disorder					0.95 (0.90–1.01)	0.092					0.94 (0.88–0.99)	0.048
Safety							1.01 (0.98–1.05)	0.448			1.03 (0.98–1.07)	0.209
Garbage collection and schools services									1.02 (0.98–1.06)	0.418	1.01 (0.97–1.04)	0.761
Random effects												
AIC	1579.70		1581.93		1580.88		1581.6		1581.61		1586.47	

*mw* minimum wages, *AIC* Akaike information criterion

N individual level = 801; N contextual level = 146; ^1^For an increase of 5 years in age

Null model: AIC = 1639.54

Table 4 presents the correlation matrix between the scales included in the multivariate model. There were a significant and positive correlation between: 1. *Walking environment* and *Safety* scales; 2. *Walking environment, Garbage collection and school services* scales; 3. *Physical Disorder* and *Safety* scales; 4. *Safety, Garbage collection and school services* scales. A significant and negative correlation encountered were between: 1. *Walking environment, Physical Disorder* scales; and 2. *Physical Disorder, Garbage collection and school services* scales.

**Table 4:** Correlation matrix between the scales

Scales	Walking environment	Physical Disorder	Safety	Garbage collection and School services
Walking environment	1			
Physical Disorder	−0.12*	1		
Safety	0.54*	0.18*	1	
Garbage collection and School services	0.50*	−0.09**	0.34*	1

*P ≤ 0.01; **P ≤ 0.05

Also, we estimated multinomial and proportional odds models using life satisfaction on an ordinal scale. However, as the original variable had a very asymmetric skewed distribution to the right, these models failed to be adequately adjusted.

## Discussion

This study aimed to investigate the association of life satisfaction with individual characteristics and objective measures of the built environment among old population living in Belo Horizonte, Brazil. The multilevel analyses showed higher prevalence of life satisfaction in old people with higher incomes, higher religious participation, who practiced physical activity and who had good/very good self-rated health. In addition, we observed that when the contextual characteristics were adjusted separately by the individual characteristics they were not significant. We also observed a lower prevalence of life satisfaction among those who lived in neighborhoods with high physical disorder levels, after adjusting for individual and other contextual characteristics such as walking environment, safety, and presence of garbage collection and school services.

Given the main objective of this paper, we will first discuss the context-level variables and then the individual-level variables. A recent study, carried out among inhabitants of urban areas in five different European countries, corroborates with our finding. This study showed that individuals who perceived their neighborhood as having lower physical disorder - for example: free from rubbish, litter and graffiti - were more likely to be satisfied with their lives [32]. No studies that investigated the association between life satisfaction and objective measures of physical disorder were found.

Neighborhood physical disorder is understood as a key to indicate that informal social control has been broken [33]. This characteristic may have deep effects on the development of neighborhood trust, attachment, and participation in community life [34–36]. In the Brazilian context, neighborhood with higher social vulnerability has higher physical disorder [24]. Within a disordered environment, many neighbors, especially older adults, may be reluctant to venture outside, reducing their ability to form ties and observe positive neighborhood interaction [37]. Additionally, the literature regarding life satisfaction and social environment among old people demonstrates that perceived social cohesion and social interaction are positively associated with life satisfaction [16, 38, 39].

Social cohesion and social interaction may influence life satisfaction of old people in several ways. First, social cohesion positively impacts the strength of relationships and social interactions, as well as collective attachment to the neighborhood, and is thus expected to enhance individuals’ life satisfactions [40]. Second, elders living in more cohesive communities may receive more instrumental and effective support [38], which are resources that can contribute to life satisfaction [41, 42]. Third, neighborhood social cohesion and social interaction may promote both physical activity [43] and greater religious participation among elders. Previous studies as well as this study show that religious participation and physical activity are positively associated with life satisfaction [14, 28], as will be discussed below.

Furthermore, previous studies report the association between objective measures of neighborhood physical disorder or self-perceived neighborhood physical disorder, and several health-related outcomes and risky health-related behaviors in adults [44–49], explained through the psychosocial processes of perceived danger and weakened social cohesion and social interaction [33].

When each contextual characteristic is adjusted for individual characteristics (models 2 to 5), they are not associated with life satisfaction. This could perhaps be explained due to the fact that the individual characteristics play a more important role in life satisfaction in the old population. In addition, the main results observed in this study (model 6) can be explained due the fact that the scales are correlated with each other.

This study did not find any association between life satisfaction and other built environment characteristics. Studies that investigated the associations between objectively assessed and perceived built environment characteristics and life satisfaction have found that living in neighborhoods with a higher percentage of streets with well-maintained green areas and residential buildings in good conditions, more water and green space or public parks, and with easy access to convenient public transportation and to cultural and leisure amenities was associated with life satisfaction [4, 16, 32, 50]. However, these studies are related to a total adult population and are not specific to older adults.

Age was found to be positively associated with higher prevalence of satisfaction with life. This result is in agreement with other studies [5, 51, 52], and different authors have postulated that this association could be happening through a socioemotional selectivity theory, suggesting that as people age, they accumulate emotional wisdom that leads to the selection of more emotionally satisfying events, friendships, and experiences [5, 53].

Also, at the individual level, we found that higher income was associated with higher prevalence of life satisfaction, corroborating the results of studies focused on non-old and old population. A study conducted by the Gallup Organization of more than 450,000 non-old adult residents in the United States demonstrated that low income was associated with low life satisfaction [54]. A research project carried out in Turkey analyzed data from 1990 to 2013 and the results showed that the prevalence of dissatisfaction was higher for low-income old people. This study also showed that in wave 2005–2009, the odds of dissatisfaction significantly decreased, showing an improvement from the previous wave, hit by a severe economic crisis in 2001 [55]. In addition, surveys developed in Central Eastern European countries and Sweden reported that life satisfaction is associated with income among the old adults [12, 27]. These studies’ findings bolster the conclusion that income is associated with life satisfaction in countries with socioeconomic characteristics similar to Brazil [5, 13, 56]. This association may be explained by the greater ability/resources of high-income people to fulfill essential and psychological needs, increasing satisfaction with one’s standard of living through the access to goods and services [57].

In this study, participation in religious services was associated with life satisfaction, indicating that old people who participate in religious activity more than twice a month report higher life satisfaction than those who do not participate or participate less often. These findings are corroborated by others studies among general adults and old populations in particular [28, 29, 58–61]. Religious activities may influence in life satisfaction for numerous reasons. First, religious communities provide social integration and support for their members and encourage them to have faith in situations of vulnerability [62]; second, religiously active individuals tend to have greater resiliency following divorce, unemployment, illness, and bereavement, recovering more quickly and more fully; and third, religious communities promote norms regarding personal lifestyles, such as interpersonal and familial relationships, and health behaviors, which could enhance an individual’s life satisfaction [63].

Physical activity was another individual-level characteristic that was associated with greater life satisfaction. Life satisfaction was more prevalent in old people who reported to practice physical activity. Cross-sectional and prospective studies examining the association between physical activity and life satisfaction in older adults have found that more active people tend to experience greater life satisfaction compared to their less active peers [64–67]. Additionally, a recent meta-analysis on this theme showed that old people exhibit a stronger association between physical activity and life satisfaction [14]. Physical activity may lead to higher life satisfaction not only due to the physiological benefits it confers, especially to functionality and physical health, but also due to the fact that it enables greater social interaction.

Finally, life satisfaction was more prevalent among participants that had a good/very good self-rated health. Other studies, in both developed and developing countries, have shown similar results among old people [15, 27, 68, 69]. Health plays an important role in life satisfaction and self-rated health provides more information about life satisfaction, as compared to medically-based health measures [15, 69]. A perceived physical vulnerability can amplify the effects of dissatisfaction in old people [70] and, moreover, the perceptions of aging itself influence and are influenced by psychological, physical, functional, clinical, and environmental dimensions [71].

One strength of our study was the use of objective measures of the built environment. This is the first study based on Latin America, to our knowledge, that investigated the built environment characteristics associated with older adults’ satisfaction, using multilevel analysis. Many researches that investigated health or other related health outcomes and urban arrangements exclusively rely on the perception of participants about the neighborhood that they live. The objective measurement of the environment can minimize the possibility of a “common source” bias [72]. Individuals’ perceptions of the environment may be influenced by personal factors. Also, individuals’ residences may be based on their health or their predisposition to given behaviors [73].

We would like to highlighted that, although the item we used to assess life satisfaction is an accepted measurement approach for this topic worldwide [74, 75], there is no gold standard measure for this construct, and self-reported measures of life satisfaction can be vulnerable to a variety of response biases [76]. For this reason, comparisons between studies are quite difficult and their interpretation should be circumspect, because life satisfaction has been measured in different ways across studies from different parts of the world. On the other hand, the administration of the SALS is simple, does not require a major investment of time for either, respondents and interviewers, and it is easily understood by participants [74]. These characteristics of the instrument are especially important in the Brazilian context where 42.3% of the population has less than 8 years of schooling [77], and even higher in the population of our study. Therefore, despite the absence of a transcultural adaptation to Portuguese or validated in the country, SALS is considered a robust measurement of the life satisfaction and its use is strongly acceptable in our context.

Furthermore, other methodological issues should be considered when interpreting the results. Data from this study, by design, are from only two health districts, namely Barreiro and Oeste. Therefore, findings may not be representative to the entire population of the city, although they are similar to others health districts in terms of demographics and socioeconomic characteristics. The BH Health Study was not designed specificaly to assess old populations and thus, for the present study, we used a subset of the sample as a whole. Consequently, conducting analyses stratified by age, as found in additional studies in the literature, is unfeasible due to the small size of the sample of participants included in the 80 years old or older group (*n* = 100), as well as the small size of the sample in some census tracts. The cross-sectional design of this study does not allow for the inference of temporality between exposures and outcomes and the results are susceptible to influence by behavioral, cultural and social factors due to the use of self-reported measures at the individual level.

At the contextual level, the measurement of some attributes may be limited as certain items are liable to temporal variation [24]. A more reliable measurement would require more than one observation for the same segment, so conditions could be averaged across different times and days of the week [24]. Additionally, the assessment of the built environment was conducted 3 years after the BH Health Study. On the other hand, the multilevel analysis approach we used, adjusted for the main individual and also for contextual factors, which are known to confound the associations researched, is considered the most appropriate for evaluating contextual characteristics. This modeling strategy allows for the examination of relative variance at different hierarchical levels and encourages the development of a research hypothesis that examines the role of context [78].

## Conclusions

Despite limitations, this analysis advances into the literature regarding built environment and life satisfaction and provides the first estimates of such associations for an old population living in an urban area of Brazil. Some individual characteristics, as well as the neighborhood physical disorder were associated with life satisfaction. Future studies should include prospective analyses and should explore multiple environmental characteristics, such as social cohesion, social interaction, and also variables of the social environment. The present findings suggest that life satisfaction should be assessed whenever evaluating urban redevelopment programs designed to improve neighborhood characteristics and to reduce physical disorder, especially among old adults.

## Data Availability

Data will not be shared. Data were from a household survey conducted by the Observatory for Urban Health in Belo Horizonte (OSUBH) at the Federal University of Minas Gerais. Currently, the authors do not have any special access privileges to these data and confirm that interested researchers may apply for access to these data in the manner described. Data supporting the conclusions of this study are included within the article.

## Abbreviations

95%CI: 95% confidence interval
AIC: Akaike information criterion
OSUBH: Observatory for Urban Health in Belo Horizonte
PR: Prevalence ratio
SALS: Self-Anchoring Ladder Scale
SD: Standard deviation
SSO: Systematic social observation
